# The prevalence and association with health-related quality of life of tungiasis and scabies in schoolchildren in southern Ethiopia

**DOI:** 10.1371/journal.pntd.0005808

**Published:** 2017-08-03

**Authors:** Stephen L. Walker, Eglantine Lebas, Valentina De Sario, Zeleke Deyasso, Shimelis N. Doni, Michael Marks, Chrissy H. Roberts, Saba M. Lambert

**Affiliations:** 1 London School of Hygiene and Tropical Medicine, London, United Kingdom; 2 Department of Dermatopathology, St. John’s Institute of Dermatology, Guy’s and St Thomas’ NHS Foundation Trust, London, United Kingdom; 3 Department of Dermatology, Centre Hospitalier Universitaire de Liège, Liège, Belgium; 4 Hospital for Tropical Diseases, London, United Kingdom; 5 Chito Health Centre, Yirgacheffe, Ethiopia; 6 Department of Dermatology, ALERT Center, Addis Ababa, Ethiopia; 7 Department of Family Medicine, Suisse Clinic, Addis Ababa, Ethiopia; Institut Pasteur, FRANCE

## Abstract

**Background:**

The prevalence of skin disease in low and middle income countries is high and communicable skin diseases are a significant public health problem. Tungiasis is an ectoparasite infestation caused by the flea *Tunga penetrans*, which has a widespread geographical distribution. Tungiasis causes painful skin lesions and may affect activities of daily living.

**Objective:**

We wished to determine the prevalence and impact of tungiasis and scabies in schoolchildren in southern Ethiopia.

**Methods:**

A cross-sectional study was performed in which students were examined by dermatologists and the skin disorders recorded. Individuals with pyogenic skin infections, scabies and tungiasis were also invited to complete the Children’s Dermatology Life Quality Index.

**Results:**

There was a high burden of skin disease amongst this cohort with more than 40% having an ectodermal parasitic skin disease. The majority of these were due to tungiasis. Tungiasis was evident in more than a third of children and was associated with onychodystophy. There was a significant association between wearing “closed” footwear and a greater number of tungiasis lesions but not tungiasis *per se*. Dermatophyte infections, acne and plantar maceration secondary to occlusive footwear were also common.

Scabies and tungiasis appeared to have a significant negative effect on quality of life.

**Conclusion:**

Tungiasis is highly prevalent in schoolchildren in the part of Ethiopia where the study was conducted and is associated with a deleterious effect on quality of life. The role of footwear in both preventing and possibly exacerbating cutaneous ailments in this setting requires further study.

## Author summary

Skin disorders are very common in all geographical locations and especially so in low and middle income countries. In these settings many of the commonest skin disorders are communicable.

Human ectoparasites such as lice and scabies mites live on the skin and *Tunga penetrans* fleas enter the skin to shed their eggs and complete their lifecycle. All of these ectoparasites cause significant morbidity particularly in poorer communities.

We have shown that the infestations of scabies and tungiasis were the commonest skin disorders in the Ethiopian schoolchildren examined in this study. These conditions are associated with a significant impact on the quality of life of these children as measured by the Children’s Dermatology Life Quality Index.

Footwear protects individuals from many conditions both infectious and non-infectious however in this cohort footwear was associated with maceration of the skin and this requires further investigation.

## Introduction

The prevalence of skin disease in low and middle income countries is high and communicable skin diseases are a significant public health problem in these settings [1, 2]. Community, school and hospital studies in Ethiopia have demonstrated a high prevalence of skin disorders in children. In a study of 112 children attending a school in the southwest of Ethiopia the ectodermal parasitic skin diseases (EPSD) scabies and pediculosis were the most common diagnoses followed by fungal, viral and bacterial skin infections[3].

Tungiasis is an ectoparasite infestation caused by the flea *Tunga penetrans*[4]. The disease occurs in the Caribbean, South America, Africa and India particularly during the hot, dry season[4, 5]. The gravid female flea burrows into the epidermis causing a nodule which increases in size up to 1 cm. The female sheds eggs into the environment over a period of approximately two weeks and the life-cycle continues. Individuals often have multiple lesions and the infestation causes a chronic, pruritic and painful, inflammatory response at the site of deposition of the eggs. Affected individuals have pain and may have difficulty with walking. Other sequelae include loss of nails, ulceration, sleep disturbance and difficulty with grip[4, 6]. Tungiasis is also a risk factor for tetanus[4]. In southern Ethiopia members of the community, school staff and governors of the Adame School in Yirgacheffe expressed concern that many children were unable to attend school for prolonged periods because of tungiasis which is known as mujale locally.

The prevalence of tungiasis in Ethiopia is uncertain. There are reports of travellers returning from Ethiopia with tungiasis and in Ethiopians who have migrated overseas[7]. A community-based study identified “young individuals” with tungiasis but did not report the prevalence[8]. A hospital based study of a paediatric cohort conducted in Tigray in northern Ethiopia did not report any cases of tungiasis[9] nor did a study of transmissible skin diseases in 1842 primary schoolchildren in the North Gondar region of northwest Ethiopia[10]. In contrast, other studies conducted in east Africa have reported a large burden of tungiasis. A household survey conducted in rural eastern Uganda reported that 22.5% of respondents were affected by tungiasis at the time of data collection and that 41.5% had been affected in the preceding month[11]. Similarly, a community based study in western Tanzania reported a prevalence of 42.5%[12].

There are few studies specifically addressing the burden of tungiasis in children. One study from Kenya showed that 19.1% of primary schoolchildren had tungiasis[13] whilst two cross-sectional studies of children attending schools in rural Nigeria reported prevalence rates of 24.4%[14] and 30.4%[15].

We conducted a study to investigate the prevalence and burden of skin disease, in particular tungiasis, amongst children attending a school in Yirgacheffe.

## Methods

### Study location

Adame School is located at an altitude of 1900m in the Yirgacheffe Woreda, Gedeo Zone approximately 10 km from Yirgacheffe town in the south of Ethiopia. Yirgacheffe has a subtropical oceanic highland climate with an average rainfall in June of 112 mm. The school has eight grades, and each grade is composed of two classes. The school day is divided into morning and afternoon sessions with children in grades 1 to 4 attending in the afternoon and children in grades 5 to 8 attending in the morning. The leadership team of the school extended an invitation to the research team to conduct the study at the school following consultation with all relevant stakeholders. It was beyond the scope of the study to involve other schools in the research.

### Participant recruitment

The study was conducted in June 2016. All students at the school were invited to participate with the exception of those in Grade 8 who had finished their final examinations.

Over three consecutive days all participating individuals had a complete skin examination performed. Examinations were conducted in a systematic way of the skin (with the exception of the genital area), hair, nails and oral cavity. The examinations were performed by pairs of examiners comprised of: one of two experienced dermatologists and either a Gedeo speaking Health Officer based in the district or an Amharic speaking Primary Care Physician. Students were brought to the waiting area class by class by their teacher and individually examined in a private setting by members of the study team of the same gender unless a third opinion was deemed necessary. The second dermatologist provided an additional opinion in cases of uncertainty and a consensus was reached. Skin diagnoses were made clinically with the aid of a dermatoscope (Heine Delta 20 Plus) where appropriate.

The case definition for tungiasis was visual confirmation of the presence of a gravid female flea in the skin or a history of tungiasis in the two weeks preceding the examination and the presence of the characteristic cutaneous crateriform pits. Scabies was diagnosed in those students with pruritus and clinical features suggestive of scabies (typical lesions in a distribution consistent with scabies)[16] or in whom *Sarcoptes scabiei* was visualised using a dermatoscope. Crusted scabies was graded using a previously published severity scale[17].

The number of siblings aged under 16 years old living in their household was recorded for each student and the number of days of absence from school in the previous four weeks was provided by the teacher from the class register. For the purposes of the study, data were only collected on students who were less than 17 years of age although older students were examined and treated if they wished. We categorised the type of footwear worn by students at the time of their examination as “none”, “open” or “closed”. “Closed” footwear enclosed the toes and provided coverage of the sole, heel and dorsum of the foot.

Students who were diagnosed with tungiasis, scabies or a pyogenic skin infection were asked to provide a specimen of urine and dipstick urinalysis using Combur 10 Test (Roche) was performed. Students diagnosed with any of these conditions were also invited to complete a Children's Dermatology Life Quality Index (CDLQI)[18]. The CDLQI was administered by a trained researcher translating directly into Gedeo from the English version.

A limited study formulary was available for the immediate treatment of certain conditions. The formulary included the following topical preparations: an emollient, antiseptic, antibiotic, antifungal, corticosteroid and scabicide. Students with more complex skin problems or non-cutaneous pathology were referred, with a comprehensive referral letter, to their Health Centre by the Health Officer who was a member of the study team. Individuals with *T*. *penetrans* in situ were advised to have them removed.

The headteacher reported that the school had 1400 pupils enrolled. We assumed a precision of 5% and a conservative estimate that the prevalence of skin disease was 20%. We calculated a sample size of 210 for 95% Confidence Interval with specified limits [15%-25%].

Data were collected anonymously directly on to Google Android devices (ASUS Nexus 7) using the ODK (Open Data Kit) application and uploaded remotely to the dedicated secure server at the London School of Hygiene and Tropical Medicine. Data were analysed using R v 3.3.0[19]. The differences between groups was assessed using Chi squared test with simulation of p value to account for small numbers for categorical and ordinal data. Odds ratios and confidence intervals were calculated using Mantel-Haenszel methods. The t test was used for continuous variables. The level for statistical significance was set at p ≤ 0.05.

### Ethics statement

Ethical approval was obtained from the Yirgacheffe Woreda and the Ethics Committee of the London School of Hygiene and Tropical Medicine (Reference: 11256). Written informed consent was provided by a parent or guardian for all participants under 18 years of age. In addition, verbal assent was obtained from all children.

## Results

Data were collected on 343 individuals from 14 classes. All pupils present in school on the day their class was examined participated in the study. 190 (55.4%) were male and the median age was 11 years (Range 5–18, IQR 9–13). At least one skin disorder was diagnosed in 252 students (73.5%). Demographic data, number of diagnoses and footwear status are shown Table 1.

**Table 1:** Demographics, number of diagnoses and footwear. IQR = Interquartile range.

	n = 343 (%)	
**Gender**	Male 190 (55.4)	Female 153 (44.6)
**Age**	Median 11 [5–18] IQR 9–13	
**Number of skin disorders**	**Number of individuals**	
0	91 (26.5)	
1	148 (43.1)	
2	83 (24.2)	
3	18 (5.2)	
4	3 (0.9)	
**Type of Footwear**		
None	8 (2.3)	
Open	168 (49.0)	
Closed	160 (46.6)	
Not recorded	7 (2.0)	

The prevalence of skin disorders is shown in Table 2. EPSD were the most common disorders (n = 139, 40.5%) followed by fungal infections.

**Table 2:** Prevalence of skin disorders diagnosed.

Diagnosis		Number (%)
**Ectodermal parasitic skin diseases**	**Total**	139 (40.5)
	**Tungiasis**	119 (34.7)
	**Scabies**	19 (5.5)
	- **Non-crusted**	17 (5.0)
	- **Crusted**	2 (0.6)
	**Pediculosis capitis**	1 (0.3)
**Fungal infections**	**Total**	93 (27.1)
	**Tinea capitis**	42 (12.2)
	**Tinea pedis**	31 (9.0)
	**Pityriasis versicolor**	13 (3.8)
	**Tinea corporis**	6 (1.7)
	**Tinea faceii**	1 (0.3)
**Pityriasis alba**		58 (16.9)
**Plantar maceration +/- pitted keratolysis**		54 (15.7)
**Acne**		49 (14.3)
**Viral infections**	**Total**	22 (6.4)
	**Viral warts**	17 (5.0)
	**Molluscum contagiosum**	5 (1.5)
**Onychodystrophy (non-tungiasis related)**		12 (3.5)
**Bacterial infections**	**Total**	13 (3.8)
	**Pyoderma**	11 (3.2)
	**Folliculitis**	1 (0.3)
	**Leprosy (possible)**	1 (0.3)
**Atopic dermatitis**		6 (1.7)
**Nevus anaemicus**		4 (1.2)
**Pityriasis amiantacea**		2 (0.6)
**Angular cheilitis**		1 (0.3)
**Digital fibroma**		1 (0.3)
**Erythrasma**		1 (0.3)
**Lymphoedema**		1 (0.3)

Scabies was diagnosed in 19 (5.5%) individuals of which two cases fulfilled the criteria for grade 1 crusted. Each case of crusted scabies was associated with four cases of non-crusted scabies in their respective classes of 37 and 31 pupils. Only five classes did not have any cases of scabies. Two cases of scabies were associated with secondary bacterial infection. The CDLQI was applied in 15 (78.9%) of the individuals with scabies and the median score was **7** (IQR 6–9).

A detailed analysis was performed of the 119 individuals with tungiasis (Table 3):

**Table 3:** Tungiasis demographics, number of lesions, distribution and complications.

**Tungiasis**		119
**Male**		75 (63.0)
**Female**		44 (37.0)
**Age**		Median 11 [7–15] IQR 9–13
**Single lesion**		2 (1.7)
**2–5 lesions or more**		37 (31.1)
**6–9 lesions or more**		30 (25.2)
**10 lesions or more**		50 (42.9)
**Location**	Fingers	5 (4.2)
	Forearm	1 (0.8)
	Buttocks	3 (2.5)
	Feet	116 (97.5)
**Complications**		
None		74 (62)
Onychodystrophy		40 (33)
Infected tungiasis		6 (5.0)
**Footwear**		
None		2 (1.7)
Open		62 (52.1)
Closed		54 (45.4)
Not recorded		1 (0.8)
CDLQI (n = 104)		Median 7 [0–16] IQR 6–9

**Fig 1:**
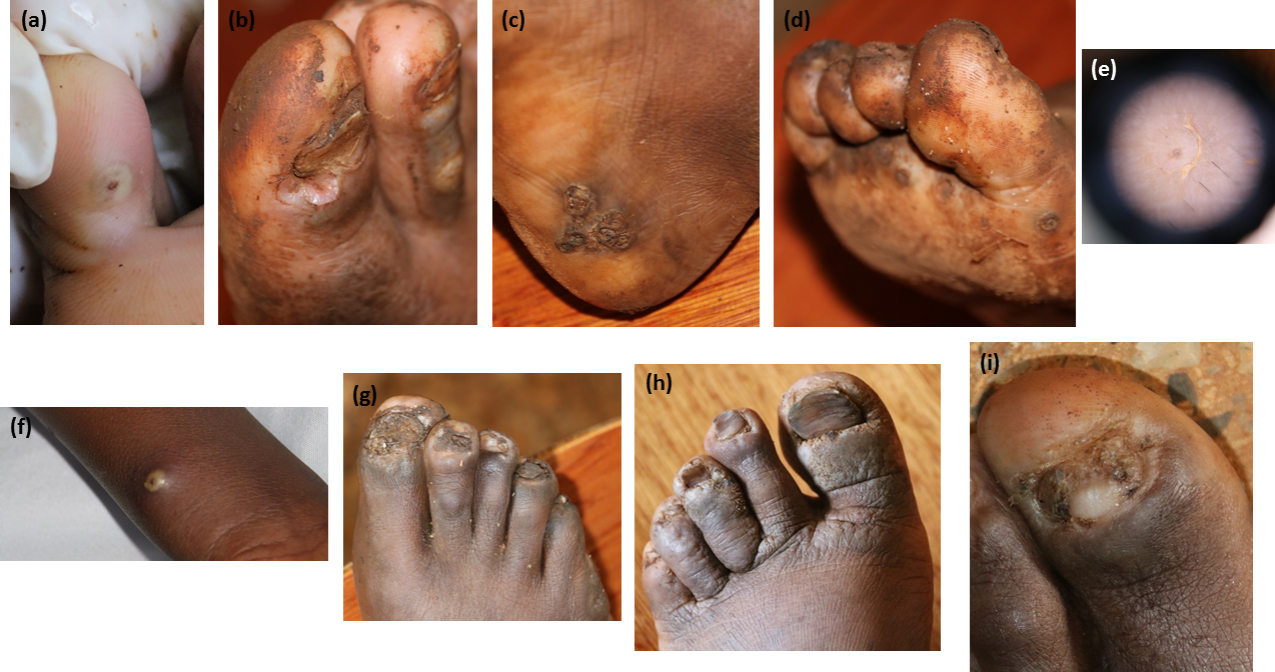
Tungiasis. a. Tunga lesion right little toe, b. two periungual lesions proximal nail fold right great toe, c. grouped tunga lesions right heel, d. eggs of *Tunga penetrans* and tunga lesions, e. dermoscopic view of tunga lesion, f. tunga lesion right forearm, g. eggs *tunga penetrans* and nail dystrophy, h. koilonychia, i. paronychial changes with partial nail loss.

Tungiasis occurred on the feet of 97.5% of individuals (Fig 1A, 1B, 1C, 1D and 1E). Only three students had tungiasis without involvement of the feet (Fig 1F). Males were more likely to be diagnosed with tungiasis (OR = 1.62 [1.02–2.54], p = 0.037). There was no significant association between the presence of tungiasis and type of footwear (OR = 1.12 [0.72–1.76], p = 0.699). Children wearing closed type footwear on the day of examination were significantly more likely to have ten or more tungiasis lesions (OR 3.44 [CI 1.6–7.4] p = 0.002).

Onychodystrophy secondary to tungiasis occurred in 33% of students. The nail changes seen were fissuring, thickening and loss of the nail plate and koilonychia (Fig 1G, 1H and 1I).

In all three individuals with tungiasis affecting the buttocks there was associated secondary bacterial infection. The CDLQI was applied in 104 (87.4%) individuals with tungiasis who had a median score of 7 (IQR 6–9).

**Fig 2:**
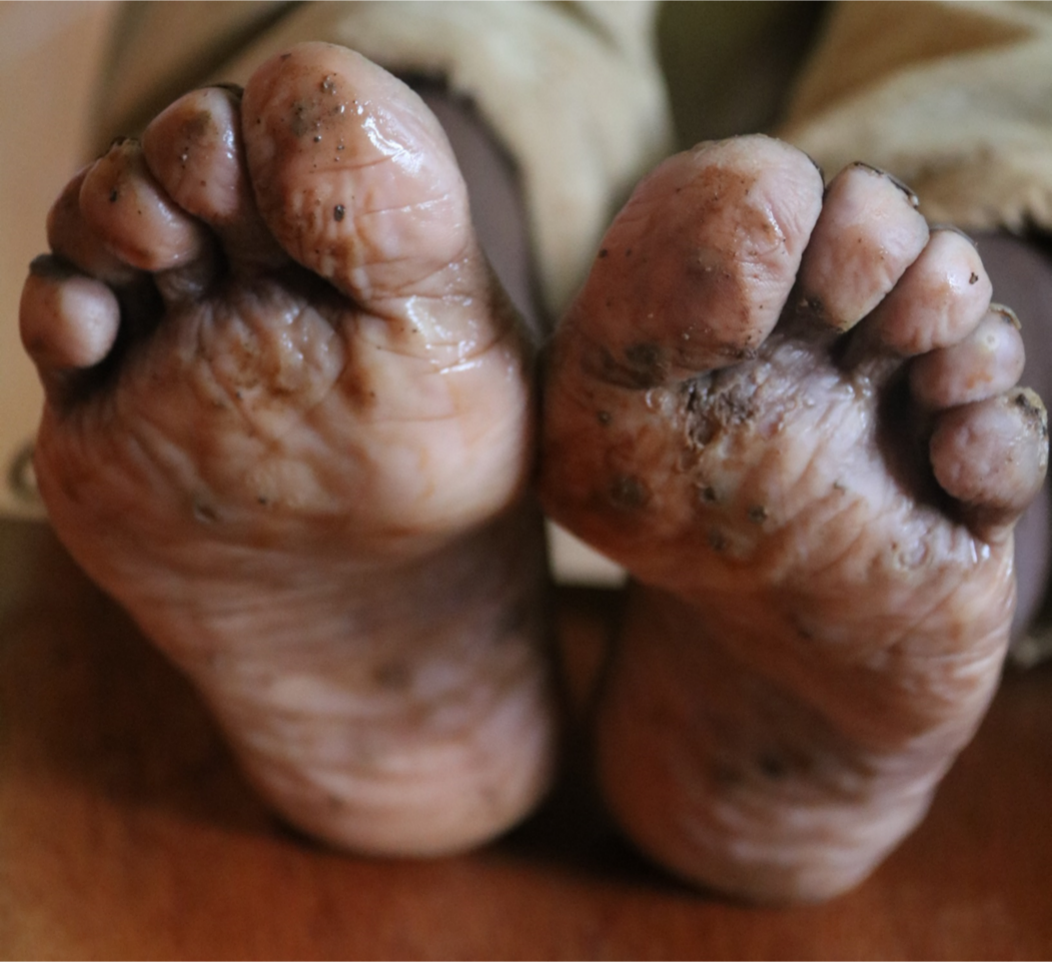
Plantar maceration and tunga lesions.

Fifty-four individuals (15.7%) had plantar maceration (Fig 2) with or without pitted keratolysis. This was typically malodorous and bilateral. The type of footwear that was observed on the day of diagnosis was significantly associated with the diagnosis of plantar maceration (P = 0.0004). Children who wore “closed” footwear were almost seven times more likely to be diagnosed with plantar maceration (OR 6.82 [3.20–14.53], p = 8.54 x 10^−8^) than children who wore either no footwear or “open” type shoes.

Urinalysis was performed in 126 (36.7%) students diagnosed with pyogenic skin infections, scabies or tungiasis of whom 17 (13.5%) had haematuria.

There was no significant association between either tungiasis (OR 0.997 [0.93–1.06], P = 0.97) or scabies (OR 0.989 [0.86–1.14] P = 0.94) and absenteeism from school. The number of siblings an individual had did not associate with diagnosis of either tungiasis (OR 0.94 [0.88–0.99], P = 0.25) or scabies (OR 1.19 [1.06–1.19], P = 0.12).

## Discussion

This study demonstrates a high prevalence of skin disorders affecting pupils attending the Adame School in Yirgacheffe, Ethiopia. The data are consistent with previous studies of schoolchildren in Ethiopia and demonstrate high levels of EPSD and fungal infections[3, 10]. Pityriasis alba and acne were also common.

The most common EPSD was tungiasis and boys were more likely to be affected than girls. More than one third of children were infested and the majority had lesions on the feet followed by the fingers and buttocks. These findings are similar to those of studies of tungiasis affecting schoolchildren in Kenya and Nigeria[14, 20]. Onychodystrophy secondary to periungual involvement by tungiasis was the most commonly identified complication. The periungual region of the feet has been previously shown to be the commonest location for lesions of tungiasis with involvement at these sites occurring in 85.7% of affected individuals in a cohort from Brazil[6]. It is not surprising that the infestation therefore leads to nail complications some of which are likely to be permanent.

Children with tungiasis had a median CDLQI of seven which is considered to show a moderate impact on quality of life[21]. Whilst this suggests that tungiasis may be associated with a clinically important impact on quality of life these findings should be interpreted with caution as the CDLQI has not been validated in this specific population nor in tungiasis. We used the English version of the questionnaire which was translated for each student and may therefore have been subject to considerable variation. Further research is warranted to explore the impact of tungiasis and other ESPDs on quality of life.

In contrast to our findings, footwear has previously been reported to reduce the risk of acquiring tungiasis[22]. In our study relatively few children were barefoot at the time of examination and this may have limited our power to detect an increased risk in this group. The use of footwear by students at school may not be a reliable indicator of footwear use at other times. We may therefore have underestimated the extent to which some children are barefoot outside of school and at risk of acquiring tungiasis. A previous study of schoolchildren in rural Ethiopia reported that 54% stated they consistently wore footwear in the three days prior to being interviewed[23].

The association between wearing closed shoes and an increased number of tungiasis lesions was unexpected and must be treated with caution due to the small sample size. It is plausible that larvae may develop from eggs shed by gravid female fleas and complete their life-cycle within the shoe resulting in an increased risk of further infestation. Previous studies have shown that, despite wearing closed shoes, soil was still adherent to the feet and the inside of shoes of 50% of children in Ethiopia[23] and other authors have emphasised the need to keep shoes and socks free from eggs and larvae[13].

Footwear has been shown to protect individuals from acquiring a wide range of diseases including soil-transmitted helminths, *Mycobacterium ulcerans* disease, cutaneous larva migrans and podoconiosis[22, 24]. In this study 15.7% of the students examined had unpleasant plantar maceration (with or without pitted keratolysis). There was a strong association of plantar maceration with the wearing of closed shoes. Plastic shoes are often purchased by Ethiopians in rural areas because they are more affordable[25] and most individuals own only one pair of shoes[25]. These factors in conjunction with the ambient temperature and rainfall and a lack of frequent changes of socks leads to occlusion of the feet in an extremely moist environment, facilitating maceration and *Corynebacterium*-associated skin infections[26]. Similar problems have been reported in healthcare workers in Africa using personal protective equipment during the management of patients with Ebola[27]. Prolonged contact of an occluded foot with water and sweat may result in reduced skin barrier function and persistent dermatitis[28].

Footwear is an important public health intervention to prevent many debilitating and disabling conditions. To minimise additional problems such as plantar maceration which may limit adherence, it is vital that individuals have access to affordable, well-fitting, robust footwear. It is also important that there is the opportunity to clean and dry footwear regularly.

The prevalence of scabies may have been reduced by the administration of ivermectin three months earlier. Yirgacheffe is endemic for lymphatic filariasis (LF) and the Ethiopian Federal Ministry of Health implements annual Mass Drug Administration (MDA) of albendazole and ivermectin to prevent cases of LF. Ivermectin MDA has been shown to be highly effective in reducing the burden of scabies in communities that have a high burden of infestation. Ivermectin is not effective against *Tunga penetrans* [29]. Nevertheless scabies outbreaks occurred in two class groups at the school. In both classes the cases appeared to be associated with an individual diagnosed with crusted scabies. The difficulty in diagnosing crusted scabies may lead to continued transmission of the infestation among classmates particularly in settings where access to treatment is limited. Single dose ivermectin (given as part of LF MDA) may not be sufficient to treat individuals with crusted scabies.

A notable proportion, 13.5%, of children with pyogenic skin infections, scabies and tungiasis had haematuria on dipstick testing of their urine. Scabies and secondary bacterial skin sepsis is a recognised risk factor for post-streptococcal glomerulonephritis[30] however it was beyond the scope of this study to investigate this further.

The high prevalence of ESPD, fungal infections and other skin disorders in communities where neglected tropical diseases (NTDs) with major skin manifestations are endemic has implications for proposals to integrate their control and management [31]. Health workers involved in skin NTDs case finding will need to be trained to recognise common skin disorders which may be difficult to distinguish from NTDs with similar clinical features. This would reduce the potential for misdiagnosis and the inappropriate use of limited resources. An approach such as this with adequate training, resources and appropriate treatment algorithms could potentially improve skin health in general by not only reducing the burden of skin NTDs in affected communities but also the prevalence of the very common skin diseases identified in this study.

This study has a number of limitations. Firstly, the study was cross-sectional in nature which limited the amount of information about skin disorders that could be obtained. Diagnoses were made clinically and there may have been variation between examiners. The prevalence of tungiasis has been shown to vary according to the seasons in Brazil[32] and we may have found an even greater prevalence had the study been conducted during the drier months. A validated translation of the CDLQI in Gedeo was not available and individuals with tungiasis often had other skin disorders which may have influenced the quality of life data we collected.

This study highlights an extremely high prevalence of tungiasis amongst these schoolchildren in rural Ethiopia. More than 40% of children had at least one ectoparasitic skin disease highlighting that these are significant public health problems in these communities. We demonstrated a possible negative association on quality of life in children with tungiasis, the first time that this has been shown. Further research on the impact of tungiasis on quality of life and the benefits of footwear are needed. Our study demonstrates the importance of diligent examination of the feet during studies of the prevalence of skin disease so that the burden of disorders that predominantly affect this site is not underestimated. Finally, assessments of the impact of footwear programmes need to consider potential adverse effects of certain types of footwear on skin and foot health.

## Supporting information

S1 Checklist STROBE checklist. (DOC)
